# Early ambulation and dhikr complementary therapies effect on intestinal peristaltic in post-open cholecystectomy patients

**DOI:** 10.1590/0034-7167-2022-0636

**Published:** 2023-12-08

**Authors:** Angga Wilandika, Nina Gartika, Ernawati Nurfarida

**Affiliations:** I Universitas Aisyiyah Bandung. Bandung, West Java, Indonesia; II Santosa Hospital Bandung Central. Bandung, West Java, Indonesia

**Keywords:** Early Ambulation, Perioperative Nursing, Complementary Therapies, Anesthesia, General, Cholecystectomy, Deambulação Precoce, Enfermagem Perioperatória, Terapias Complementares, Anestesia Geral, Colecistectomia, Deambulación Temprana, Enfermería Perioperatoria, Terapias Complementarias, Anestesia General, Colecistectomía

## Abstract

**Objectives:**

to analyze and determine the effect of a combination intervention of early ambulation and dhikr therapy on intestinal peristaltic recovery in post-open cholecystectomy patients.

**Methods:**

a pre-experimental design with one group pre and post-test design was used. The samples were 15 post-open cholecystectomy patients which were selected using the purposive sampling technique. The data were collected using the instrument observation sheet and analyzed using the Wilcoxon test. Early ambulation used standard operational procedure in the hospital and dhikr therapy was carried out at 2 hours post-operation for 10-15 minutes.

**Results:**

there was an effect of early ambulation and dhikr therapy on intestinal peristaltic recovery in post-open cholecystectomy patients with general anesthesia (Z=-3.442; p=0.001).

**Conclusions:**

a combination of early ambulation and dhikr therapy can be recommended as interventions to improve intestinal peristaltic in a post-open cholecystectomy patient with general anesthesia.

## RESUMO

**Objetivos:**

analisar e determinar o efeito de uma intervenção que combinou deambulação precoce e terapia dhikr na recuperação peristáltica intestinal de pacientes que foram sujeitos a colecistectomia aberta.

**Métodos:**

um delineamento pré-experimental foi utilizado com um grupo pré e pós-teste. As amostras incluíram 15 pacientes sujeitados a colecistectomia aberta e selecionados por amostragem intencional. Os dados foram coletados por fichas de observação do instrumento e analisados pelo teste de Wilcoxon. A deambulação precoce utilizou o procedimento operacional padrão no hospital e a terapia dhikr foi realizada por 10-15 minutos, duas horas após a operação.

**Resultados:**

a deambulação precoce associada a terapia dhikr afetou a recuperação peristáltica intestinal de pacientes que foram sujeitos a colecistectomia aberta com anestesia geral (Z=-3,442; p=0,001).

**Conclusões:**

a combinação de deambulação precoce e terapia dhikr pode ser recomendada como uma intervenção para melhorar o movimento peristáltico intestinal de pacientes após colecistectomia aberta com anestesia geral.

## RESUMEN

**Objetivos:**

analizar y determinar el efecto de una intervención que combinó la deambulación temprana y la terapia dhikr sobre la recuperación peristáltica intestinal de pacientes sometidos a colecistectomía abierta.

**Métodos:**

se utilizó un diseño preexperimental con un grupo pretest y postest. Las muestras incluyeron 15 pacientes sometidos a colecistectomía abierta y seleccionados mediante muestreo intencional. Los datos se recopilaron por medio de fichas de observación del instrumento y se analizaron mediante la prueba de Wilcoxon. La deambulación temprana utilizó el procedimiento operativo estándar en el hospital y la terapia dhikr se realizó durante 10 a 15 minutos, dos horas después de la operación.

**Resultados:**

la deambulación temprana asociada con la terapia dhikr afectó la recuperación peristáltica intestinal de los pacientes que se sometieron a colecistectomía abierta con anestesia general (Z =-3,442; p=0,001).

**Conclusiones:**

la combinación de la deambulación temprana con la terapia dhikr puede recomendarse como una intervención para mejorar el movimiento peristáltico intestinal de los pacientes después de una colecistectomía abierta con anestesia general.

## INTRODUCTION

Gallstones are the one of most common abdominal diseases for the hospital admission. In Indonesia, estimation has shown that the occurrence of the disease is lower compared to its prevalence in the West. However, with the presence of a sedentary lifestyle trend, there is a possibility in the future that stone cases of bile will be a serious health problem in the country^(1)^. This problem deserves attention, especially in high-risk groups called ‘4Fs’, which include Fat, Female, Forty, Fair, Fertile, and Family history^(2)^.

Cholecystectomy is a surgical procedure in patients with acute cholelithiasis or gallstones, consisting of open cholecystectomy and laparoscopic cholecystectomy. It is an open operation, where the recovery of postoperative gastrointestinal function is longer than that of laparoscopic cholecystectomy. There are 14 types of anesthesia used in cholecystectomy surgery general anesthesia, spinal anesthesia, and induction block^(3, 4)^.

Postoperative gastrointestinal dysfunction (POGD), commonly referred to as postoperative ileus (POI), is a popular complication characterized by a transient impairment of gastrointestinal (GI) function after abdominal surgery^(5)^. Nausea, vomiting, abdominal tenderness and distention, absence of normal bowel sounds, and/or delay in the passage of flatus and stool are some of the signs and symptoms associated with POI^(6)^. The incidence of abdominal bloating and distension is usually among the risks that occur in postoperative day 0 patients. Impaired ambulation due to acute complications such as surgical wound pain, nausea, vomiting, and hypotension can also limit or delay the patient’s ambulation. Furthermore, the presence of a tube attached to the patient’s body such as a drain, dower catheter, and NGT limits the movement^(7)^.

Several studies have proven that early ambulation is effective for restoring intestinal peristaltic. This is because early ambulation carried out at 6 hours postoperatively has the effect of restoring intestinal peristalsis^(8)^. Dhikr is a verbal ritual, where there is a process of recitation with the appreciation of meaning. The mechanism of dhikr is the repeated pronunciation of *lafadz,* where movement occurs between teeth, tongue, and gums, which involves the jaw muscles and the temporomandibular joint. Motor activity when reciting repeatedly will stimulate gastrointestinal hormones to secrete saliva and increase gastric secretion thereby stimulating intestinal motility^(9)^.

There is still limited information on the effect of dhikr on the recovery of intestinal peristalsis, but several investigations have examined the effectiveness of dhikr on physical well-being. A previous study showed that dhikr by reciting the sentences *hauqollah, tasbih,* and *tahmid* 33 times from each sentence has the effect of reducing anxiety in preoperative patients. This proves that dhikr influences physical well-being^(10)^.

The role of nurses in postoperative patients is to meet basic needs and prevent post-anesthesia complications^(11)^. The intervention method involves a physical and spiritual approach, which is used to reduce anxiety levels and postoperative stress^(9)^.

## OBJECTIVES

To identify the effect of the combined intervention of early ambulation and dhikr on intestinal peristalsis in post-open cholecystectomy patients with general anesthesia. The results are expected to be used for the development of nursing science, which is an independent intervention in carrying out nursing care with a spiritual approach.

## METHODS

### Ethical aspects

Free and Informed Consent was obtained from all participants in the study through a written, signed, and dated informed consent form. Participants voluntarily confirm their willingness to participate in a particular trial, after having been informed of all aspects of the trial that are relevant to the subject’s decision to participate and the right to withdraw. Several methods are used to maintain participants’ confidentiality. Personal details are not written on reports, participants will be referred to by an assigned number rather than their name, and data will be stored and disposed of securely. Participants were debriefed at the end of the study. These considerations were taken into account to prevent as much harm as possible whether it be emotional, physical, or psychological.

### Design, period and place of study

This is a quantitative study using a pre and post-test experimental approach with one group design guided by the STROBE tool, in the period from January to February 2020, carry out at Ruby Timur Santosa Hospital Bandung Central, Bandung, West Java, Indonesia.

### Population or sample; inclusion and exclusion criteria

The population of this study consisted of 35 patients who were passing through cholecystectomy in Santosa Hospital Bandung Central, Bandung, West Java, Indonesia. The samples were 15 patients selected using the purposive sampling technique and the inclusion criteria were respondent post operation laparotomy cholecystectomy with general anesthesia, moslem, and ability to communicate. Meanwhile, the exclusion criteria were respondents with complication post operation such as tachycardia, hypothermia, hemorrhage, severe nausea (PONV), and those referred to the intensive care unit.

### Study protocol

The group was observed for intestinal peristalsis before and after early ambulation and dhikr intervention was carried out. Nursing interventions were carried out by practicing deep breathing, leg exercises, hands, knees, and feet, as well as changing the left and right tilt position of the patient for 30 minutes, at 2 hours postoperative, which was repeated at 6 hours.

A combination of ambulation and dhikr therapy was given at 2 hours postoperative in post-laparotomy patients and repeated at 6 hours postoperatively. Early ambulation exercises for 10-15 minutes, starting at 2 hours postoperatively were based on the relevant journals and were discussed according to the attached procedure.

The first approach started with deep breathing techniques 5 times. The exercise was continued by training the arms by moving the shoulders in abduction and rotation as well as moving the fingers, which is repeated 5 times on each hand. Leg exercises were carried out in post-laparotomy patients under general anesthesia starting with: (1) moving the leg by bending and raising the knee, followed by holding for a few seconds, lower and straightening the knee again; (2) repeating the movement every 5 times for each leg; (3) rotational movements were performed by bending the legs inward, outward, and closer to each other; (4) repeat this movement 5 times on both legs. Moreover, exercises to change the left tilted position were carried out by lying down, at 6 hours postoperatively (1) the patient is tilted for 5 minutes to the left, followed by another 5 minutes to the right; (2) this exercise can be repeated every 2 hours.

After ambulation, the patient is guided in dhikr to increase relaxation by reciting the sentence Al-Baqiyatu Ash-Salihah (*tasbih, tahmid, tahlil, takbir*, and *Al-hauqallah*) 33 times from each sentence. Recitation of dhikr was carried out after early ambulation exercises. Subsequently, peristaltic auscultation for 1 minute in the right upper quadrant of the abdomen was performed using a stethoscope as a post-test.

### Analysis of results and statistics

The sample’s sociodemographic characteristics including age, gender, education, and intestinal peristaltic frequency were displayed using descriptive statistics. A Wilcoxon test was computed to examine the mean difference in intestinal peristaltic frequency before and after the intervention. Statistical analysis was performed with a statistical software package.

## RESULTS

Based on the results as shown in Table 1, 80.0% of the respondents involved were aged 36-40 years, where 53.3% were females. Approximately 46.7% of the respondents had an education level and graduated from high school. Furthermore, there was no relationship between the patient’s sociodemographic characteristics, namely age, gender, and education with the frequency of intestinal peristalsis (final score) of the patient, with a p > 0.05.

**Table 1:** Sociodemographic characteristics and their relationship to intestinal peristaltic frequency (post-test)

Sociodemographic	Frequency (f)	Percentage (%)	Correlation (p-value) with intestinal peristaltic frequency
Age			0.284^a^
25-35	2	13.3	
36-40	12	80.0	
>40	1	6.7	
Gender			0.910^a^
Female	8	53.3	
Male	7	46.7	
Education			0.123^a^
Primary school	4	26.7	
Secondary school	7	46.7	
College	4	26.7	

^a^ *Spearman-rank test.*

Table 2 shows an increase in the frequency of intestinal peristaltic in postoperative respondents 2 hours after the intervention of a combination of early ambulation and dhikr. The mean frequency of intestinal peristaltic in all respondents before and after the intervention was 0.87 ± 0.74 and 6.27 ± 1.16, respectively. It was also discovered that the combined intervention of early ambulation and dhikr had a significant effect on the frequency of intestinal peristaltic (Z = -3.442, P <0.05).

**Table 2:** Differences in intestinal peristaltic frequency after intervention

	Intestinal Peristaltic Frequency			Z	Sig. (p value)
	Average (mean ± SD)	Min	Max		
Intestinal peristaltic frequency (pre-test)	0.87 ± 0.74	0	2	−3.442	0.001
Intestinal peristaltic frequency (post-test)	6.27 ± 1.16	5	8		

## DISCUSSION

A combination of early ambulation and dhikr therapy has increased intestinal peristalsis in patients after cholecystectomy surgery with general anesthesia. The results showed that all respondents experienced intestinal peristaltic recovery at 2 hours postoperatively. The action mechanism of early ambulation on intestinal peristaltic activity such as exercise can stimulate the parasympathetic nerves to the intestinal muscles, leading to a wave of intestinal motility, with the work of the parasympathetic nerves. This will also cause the release of acetylcholine, thereby increasing the conduction of active waves along the intestinal wall, which can stimulate intestinal peristalsis and accelerate flatus^(12)^. The mechanism of repeated dhikr will affect the central nervous system to release serotonin, norepinephrine, and endocannabinoid hormones, which will trigger relaxants in all organs. This will activate all organs’ vasodilators, especially in the intestine, and the enteric nervous system (ENS) in the myenteric plexus to stimulate the release of peristaltic waves^(13)^.

The success of early ambulation in accelerating the recovery of postoperative intestinal peristalsis has been demonstrated in previous studies. It was also discovered that the recovery of intestinal peristalsis was faster in the treatment group compared to the control^(7)^. However, this study proved that the recovery of intestinal peristalsis was faster in patients who had early ambulation and dhikr. This is triggered by some postoperative patients who experience emotional changes and had difficulty mobilizing early because concerns about pain can be helped by dhikr as an intervention with a spiritual approach^(14)^.

The dhikr technique in this study is in form of a recitation of *Albaqiyatu Ash-sholihat* dhikr 33 times. Based on the physiological aspect, relaxation during dhikr increases the work of the parasym-pathetic nerves by inhibiting the work of the sympathetic nerves. The sympathetic nervous system works through the activation of the adrenal medulla to increase the release of epinephrine, norepinephrine, as well as cortisol and reduce nitric oxide^(8)^. This situation can cause changes in the body’s response, increase metabolism, and dilate blood vessels of all organs, especially in the gastrointestinal system. This activates the nervous system in the gastrointestinal tract and stimulates the emergence of intestinal peristaltic^(15)^

### Study limitations

The limitation of this study is the small number of samples, hence the results cannot represent the entire population.

### Contributions to the fields of Nursing, Health or Public Policy

The results are expected to be used for the development of nursing science, which is an independent intervention in carrying out nursing care with a spiritual approach.

## CONCLUSIONS

The results show that there is a significant effect of the combined intervention of early ambulation and dhikr on the recovery of intestinal peristaltic in post-open cholecystectomy patients with general anesthesia. This study provides important information for nurses’ reference in carrying out interventions in a combination of early ambulation with Dhikr therapy can relieve patients’ negative emotions, reduce postoperative pain, and thus accelerate the recovery of intestinal peristaltic in post-open cholecystectomy patients. Early ambulation and dhikr intervention enrich nursing interventions focusing on physical and spiritual aspects, especially in post-open cholecystectomy patients with general anesthesia. The study results also become the basis for making standard operating procedures developed by policyholders and applied to patients who experience the same problem.
